# Functional In Vitro Assessment of VEGFA/NOTCH2 Signaling Pathway and pRB Proteasomal Degradation and the Clinical Relevance of Mucolipin TRPML2 Overexpression in Glioblastoma Patients

**DOI:** 10.3390/ijms23020688

**Published:** 2022-01-08

**Authors:** Giorgio Santoni, Consuelo Amantini, Massimo Nabissi, Antonietta Arcella, Federica Maggi, Matteo Santoni, Maria Beatrice Morelli

**Affiliations:** 1 School of Pharmacy, Section of Experimental Medicine, University of Camerino, 62032 Camerino, Italy; massimo.nabissi@unicam.it; 2 School of Biosciences and Veterinary Medicine, University of Camerino, 62032 Camerino, Italy; consuelo.amantini@unicam.it (C.A.); federica.maggi@uniroma1.it (F.M.); 3 Neuropathology Laboratory, Istituto di Ricovero e Cura a Carattere Scientifico Neuromed, 86077 Pozzilli, Italy; arcella@neuromed.it; 4 Department of Molecular Medicine, Sapienza University, 00185 Rome, Italy; 5 Oncology Unit, Macerata Hospital, 62100 Macerata, Italy; mattymo@alice.it

**Keywords:** mucolipin, TRPML2, glioblastoma, VEGFA, Notch2, pRB, overall survival

## Abstract

Glioblastoma (GBM) is the most malignant glioma with an extremely poor prognosis. It is characterized by high vascularization and its growth depends on the formation of new blood vessels. We have previously demonstrated that TRPML2 mucolipin channel expression increases with the glioma pathological grade. Herein by ddPCR and Western blot we found that the silencing of TRPML2 inhibits expression of the VEGFA/Notch2 angiogenic pathway. Moreover, the VEGFA/Notch2 expression increased in T98 and U251 cells stimulated with the TRPML2 agonist, ML2-SA1, or by enforced-TRPML2 levels. In addition, changes in TRPML2 expression or ML2-SA1-induced stimulation, affected Notch2 activation and VEGFA release. An increased invasion capability, associated with a reduced VEGF/VEGFR2 expression and increased vimentin and CD44 epithelial-mesenchymal transition markers in siTRPML2, but not in enforced-TRPML2 or ML2-SA1-stimulated glioma cells, was demonstrated. Furthermore, an increased sensitivity to Doxorubicin cytotoxicity was demonstrated in siTRPML2, whereas ML2-SA1-treated GBM cells were more resistant. The role of proteasome in Cathepsin B-dependent and -independent pRB degradation in siTRPML2 compared with siGLO cells was studied. Finally, through Kaplan-Meier analysis, we found that high TRPML2 mRNA expression strongly correlates with short survival in GBM patients, supporting TRPML2 as a negative prognostic factor in GBM patients.

## 1. Introduction

Glioblastoma (GBM) is the most common type of primary brain cancer characterized by poor prognosis due to the rapid progression, active angiogenesis, enhanced tumor cell invasion and the emergence of resistance toward conventional therapy [1]. Several recent reports have clearly evidenced an emerging role for endolysosomal TRPML mucolipin channels in the GBM development [2,3,4]. The TRPML mucolipin channels belong to the transient receptor potential (TRP) superfamily of ion channels [5]. In mammals, there are three TRPML proteins (TRPML1, TRPML2 and TRPML3) encoded by *MCOLN1-3* genes [6]. The TRPML channels are six transmembrane-spanning proteins that consist of cytosolic N- and C-termini, and a pore-loop domain between S5 and S6. Human TRPML2 mRNA is expressed in the lungs, stomach, colon, mammary gland and brain [2,7,8]. Recently, a role of the TRPML2 channel in innate immune responses and in the susceptibility to bacterial and viral infections has been demonstrated [9,10]. Human TRPML2 channels localize in endosomal and lysosomal compartments, although functional activity has also been reported at the plasma membrane [11]. In addition, TRPML2 forms homo- and hetero-multimers with TRPML1 and/or TRPML3 [5,12]. An important role has been proposed for TRPML2 in trafficking and regulation along the clathrin-independent Arf6-associated endocytic pathway [13]. Human TRPML2 is a Ca^2+^-permeable non selective cation channel, which is inhibited by low extracytosolic pH and activated by phosphatidilinositol 3,5 biphosphate (PI(3,5)P2), a low abundance endolysosome-specific phosphoinositide [14]; recently a specific agonist, ML2-SA1 (EVP-22), highly selective for the human TRPML2 calcium channel with no significant activity for TRPML1 and TRPML3 channels, has been developed [15].

An association between TRPML2 expression and cancer has been reported. In pediatric acute lymphoblastic B-leukemia, the *MCOLN2* gene has been found to be hypermethylated in the 5′ regulatory region and downregulated [16]. The Human Protein Atlas reports the expression of TRPML2 mRNA and protein in colon glandular cells and in colorectal (CRC) cancers. A significant correlation between the rs9929218 variant of the cadherin-1 (CDH1), *MCOLN2* and CRC susceptibility has been demonstrated [17]. Analysis of the transcriptome in CRC showed that TRPML2 is dramatically downregulated compared with normal tissue [18]. In HN31 oral cancer cells, an increased TRPML2 expression has been evidenced [19]. In breast cancer, Huang et al., identified a gene signature associated with clinical ER and HER2 phenotypes. A 16-gene signature, including the Wnt/β−catenin signaling pathway and the *MCOLN2* gene, was found to be associated with cancer recurrence, metastasis and distinct survival patterns in breast cancer patients [20]. Moreover, recently a role of TRPML2 in prostate cancer progression via the IL-1β/NF-κB pathway has recently been reported [21]. Finally, we have previously reported that TRPML2 is expressed in human glioma tissues and its expression increases with the pathological grade [2]. Downregulation of the TRPML2 channel impaired survival and proliferation, as well as triggered DNA damage and apoptosis, through caspase-3 activation and the blockade of Akt and Erk1/2 phosphorylation, suggesting a pro-tumorigenic role for TRPML2 in glioma progression [2].

Key features of tumor growth and development are angiogenesis and invasion [22]. Among proangiogenic factors, VEGF and its main receptor VEGFR2 play a pivotal role in the regulation of tumor vessel formation making this pathway a promising molecular target for anti-angiogenic therapy [23].

Notch signaling also plays an important role in development and glioma genesis [24] promoting the generation of astrocytes from neuronal precursor cells. The Notch system is composed of four receptors (Notch1–4) and at least five ligands from the families Delta and JAG/Serrate (DSL) (JAG1, JAG2, Delta-like (Dll)-1, Dll-3, and Dll-4) [25,26,27]. Notch receptors have been demonstrated to be activated in gliomas by gain- and loss of-function studies in vitro and in vivo [28]. Notch signaling is important in GBM proliferation, differentiation and apoptosis. It is also involved in regulating responses to hypoxia and angiogenesis, which are typical features of tumors such as GBM [29,30,31].

Finally, the deregulation of the RB/E2F pathway through genetic or epigenetic changes frequently occurs in GBMs [32]. The RB/E2F pathway coordinates several important processes including cell migration and differentiation, angiogenesis and development, apoptosis and mitosis, drug-resistance and repair and cell cycle checkpoints [33]. 

The aim of our work was to study the VEGFA/Notch2 signaling pathway and the role of TRPML2 channels by using silencing/enforced and activated TRPML2 expression in migration/invasion, drug-resistance and retinoblastoma protein (pRB) degradation. Moreover, the clinical correlation between TRPML2 overexpression and overall survival in GBM patients has been evaluated. 

## 2. Results

### 2.1. Silenced or Enforced TRPML2 Expression and TRPML2 Activation in T98 and U251 Cells

The functional role of TRPML2 in GBM was evaluated by silencing and enforcing TRPML2 expression as well as by triggering TRPML2 with the specific agonist, ML2-SA1 [15], in T98 and U251 cell lines previously characterized for TRPML2 expression [2].

For silencing experiments, TRPML2 (siTRPML2) and siCONTROL non-targeting siRNA (siGLO) were used. A marked reduction of TRPML2 mRNA was evidenced in siTRPML2 compared with siGLO T98 and U251 cells (Figure S1A). The reduced expression of TRPML2 was confirmed at protein level by Western blot analysis (Figure S1B). In some experiments, to study the role of TRPML2 in more depth, the channel has been also overexpressed. In such experiments, T98 and U251 cells were transfected with pCMV-TRPML2 or pCMV vectors. A marked enhancement of TRPML2 mRNA was evidenced in pCMV-TRPML2 compared with pCMV T98 and U251 cells (Figure S2A). The increased expression of TRPML2 was confirmed at protein level by Western blot analysis (Figure S2B). Finally, for activation, the effect of the treatment with ML2-SA1 at different doses (1–50 μM) for 24 h in T98 and U251 cells was firstly evaluated. No cytotoxic effects were evidenced until 30 μM dose in both GBM cell lines (Figure S3; therefore, the 10 and 30 μM doses were used in all the experiments.

### 2.2. Gene Expression Profile in TRPML2 Silenced T98 and U251 Cells

Gene profiles of control (siGLO) and siTRPML2 T98 and U251 cells at 48 h post transfection were evaluated by Digital Droplet PCR (Table 1 and Table 2). 

We found that VEGFA mRNA expression was reduced whereas VEGFB mRNA was slightly increased in both siTRPML2 T98 and U251 cells compared with siGLO cells. NOTCH2 mRNA expression was reduced in siTRPML2 T98 and U251 cells, compared with siGLO cells. STAT3 and SPARC mRNA expression were increased in both siTRPML2 cell lines, compared with siGLO cells. Finally, among the mRNA expression of the epithelial-mesenchymal transition (EMT) markers, vimentin was increased in both siTRPML2 T98 and U251 cells, whereas the CD44 was enhanced in T98 cells, compared with siGLO cells. No significative changes in the mRNA expression of the hedgehog markers (SHH, DHH, IHH, PTCH1/2, ZEB1/2 and SMO); EPCAM and POU5F1B werealso observed in siTRPML2 T98 and U251 cells, compared with siGLO cells.

### 2.3. The VEGFA/VEGFR2 Signaling Pathway in TRPML2-Silenced, -Enforced and ML2-SA1-Activated T98 and U251 Cells

**Figure 1:**
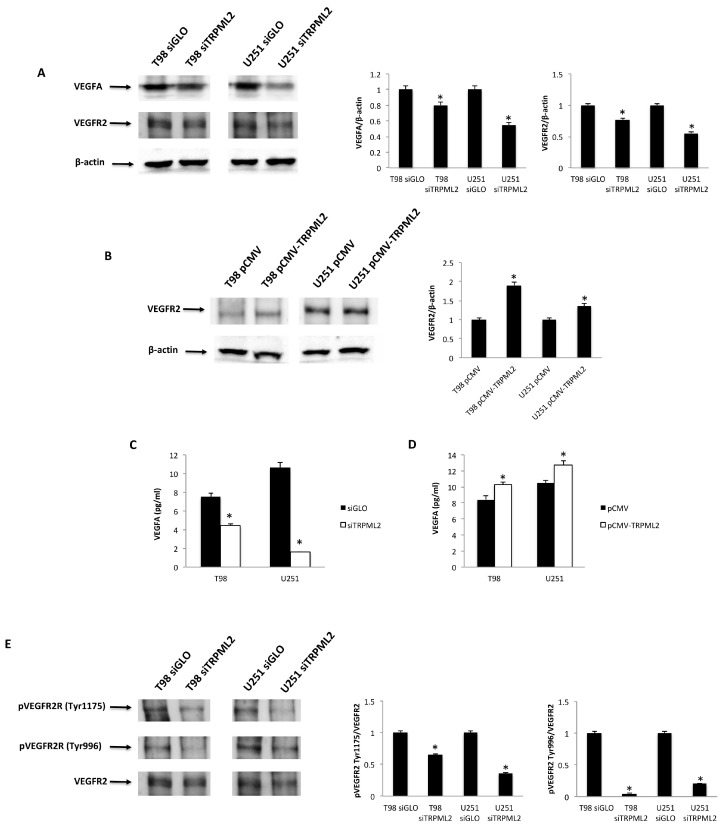
TRPML2 levels influenceVEGFR2 and VEGFA protein levels, as well as VEGFA release in T98 and U251 cell line. (**A**) Representative immunoblots reflecting VEGFA and VEGFR2 protein levels in siGLO and siTRPML2 T98 and U251 cell lines. Blots are representative of one of three separate experiments. Densitometry values were normalized to β-actin, which was used as a loading control. Data of siGLO vs. siTRPML2 T98 and U251 cell lines are expressed as the mean ± SE of three separate experiments. * *p* < 0.05. (**B**) Representative immunoblots reflecting VEGFR2 protein levels in pCMV and pCMV-TRPML2 T98 and U251 cell lines. Blots are representative of one of three separate experiments. Densitometry values were normalized to β-actin, which was used as a loading control. Data of pCMV vs. pCMV-TRPML2 T98 and U251 cell lines are expressed as the mean ± SE of three separate experiments. * *p* < 0.05. (**C**,**D**) Levels of VEGFA release measured by ELISA were evaluated at 24 in siGLO and siTRPML2 and pCMV and pCMV-TRPML2 T98 and U251 cell lines. Data are expressed as the mean ± SE of three separate experiments. * *p* < 0.05. (**E**) Representative immunoblots reflecting the activated pVEGFR2(Tyr1175) and pVEGFR2(Tyr996) protein levels in siGLO and siTRPML2 T98 and U251 cell lines. Blots are representative of one of three separate experiments. Densitometry values were normalized to VEGFR2 total levels. Data are expressed as the mean ± SE of three separate experiments. * *p* < 0.05.

The VEGFA/VEGFR2 signaling pathway is implicated in proliferation, migration and angiogenesis [34,35,36]. Thus, the expression of VEGFA and VEGFR2 was analyzed at protein level in siGLO and siTRPML2 T98 and U251 cells by Western blot (Figure 1A). Silencing of TRPML2 inhibited VEGFA as well as VEGFR2 protein expression with respect to the control cells. On the other hand, an increased VEGFR2 protein expression was evidenced in enforced pCMV-TRPML2 compared with pCMV T98 and U251 cells (Figure 1B). In addition, the effect of TRPML2 expression modulation on VEGFA release was evaluated by ELISA. Reduced and enhanced VEGFA protein release at 24 h after transfection was evident in siTRPML2 vs. siGLO and pCMV-TRPML2 vs. pCMV T98 and U251 cells, respectively (Figure 1C). 

To explore the VEGF-induced signaling, the phosphorylation status of VEGFR2 in T98 and U251 cells and the potential effects of silencing or enforcing TRPML2 expression or ML2-SA1 stimulation were studied. Phosphorylation at Tyr996 and Tyr1175 of the VEGFR2 sites evidenced at basal level in T98 and U251 cells were reduced in siTRPML2 cells (Figure 1D). Instead, enforced TRPML2 expression did not induce significant changes in VEGFR2 phosphorylation status in both cell lines, compared with pCMV control cells (data not shown). 

**Figure 2:**
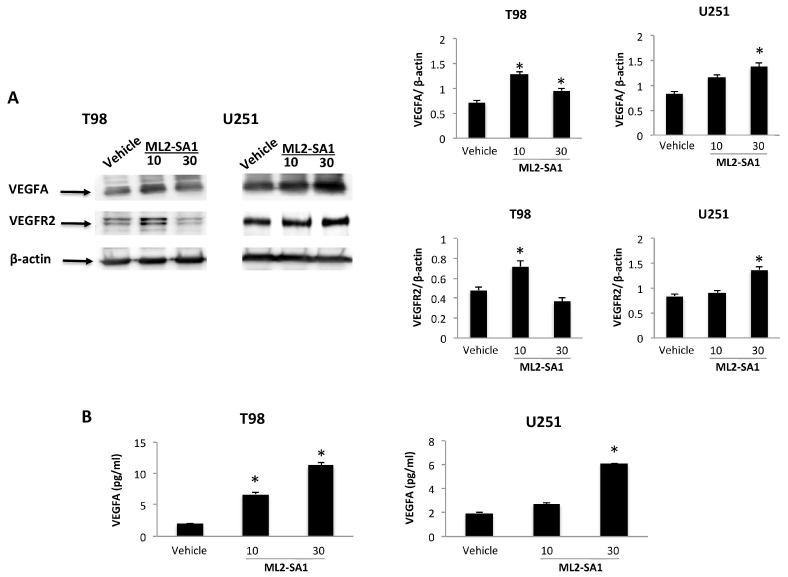
ML2-SA1 improves VEGFA and VEGFR2 proteins expression and increases VEGFA release in T98 and U251 cell lines. (**A**) Representative immunoblots reflecting VEGFA and VEGFR2 protein levels in vehicle- and ML2-SA1 (10 and 30 μM)-treated T98 and U251 cell lines. Blots are representative of one of three separate experiments. Densitometry values were normalized to β-actin, which was used as a loading control. Data are expressed as the mean ± SE of three separate experiments. * *p* < 0.05. (**B**) Levels of VEGFA release measured by ELISA were evaluated at 24 h in vehicle- and ML2-SA1 (10 and 30 μM)-treated T98 and U251 cell lines. Data are expressed as the mean ± SE of three separate experiments. * *p* < 0.05.

Then, the effects of 24 h treatment with the TRPML2 agonist, ML2-SA1 at 10 and 30 μM, on VEGFA and VEGFR2 protein levels, as well as on VEGFA release, were evaluated. Results indicated that the exposure of T98 and U251 cells to ML2-SA1 increases the VEGFA and VEGFR2 protein levels (Figure 2A), with respect to vehicle-treated cells. In addition, an increased ML2-SA1-dependent VEGFA protein release, compared with vehicle-treated T98 and U251 cells, with a maximal release at 30 μM was evidenced (Figure 2B). No changes in VEGFR2 Tyr-phosphorylation status were observed in ML2-SA1-treated T98 and U251 cells, compared with the control (data not shown).

### 2.4. Crosstalk between Notch2 Signaling and the TRPML2 Channels in GBM Cell Lines

**Figure 3:**
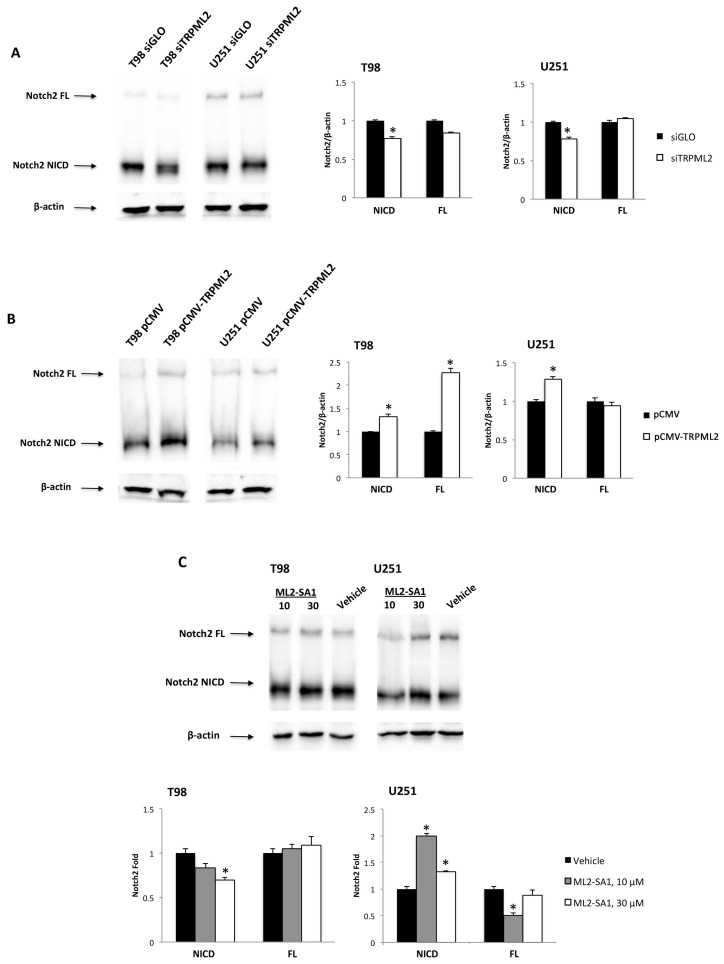
Expression of Notch2 protein in TRPML2 silenced or overexpressed and ML2-SA1 treated cell lines. Representative immunoblots reflecting Notch2 FL (full-length) and NICD (Notch intracellular domain) protein levels in siGLO and siTRPML2. (**A**) and in pCMV and pCMV-TRPML2. (**B**) T98 and U251 cell lines after 48 h post-transfection. (**C**) The Western blot analysis of Notch2 protein levels in T98 and U251 cell lines treated with vehicle or ML2-SA1 (10 and 30 μM) for 24 h. Blots are representative of one of three separate experiments. Densitometry values were normalized to β-actin, which was used as a loading control. Data are expressed as the mean ± SE of three separate experiments. * *p* < 0.05 siTRPML2 vs. siGLO cells, pCMV-TRPML2 vs. pCMV and ML2-SA1 vs. vehicle.

Gene expression profiling evidenced the reduction in Notch2 mRNA levels in TRPML2 silenced T98 and U251 cells (Table 1 and Table 2). Thus, the expression of Notch2 protein was evaluated in siGLO and siTRPML2 GBM cells by Western blot. Expressions of both the full-length (FL) and the active form of Notch2, the intracellular domains of Notch2 (NICD), were found in the T98 and U251 cell lines, suggesting that the Notch2 signaling pathway is basally activated in GBM cell lines (Figure 3A). Moreover, the silencing of TRPML2 reduces NICD Notch2 protein levels, compared with siGLO T98 and U251 cells (Figure 3A); in contrast, enforced TRPML2 expression by pCMV-TRPV2 transfection (Figure 3B) increased the expression of NICD Notch2 protein, compared with T98 and U251 control cells. Finally, treatment of U251 glioma cells for 24 h with both doses of ML2-SA1 increased the expression of the Notch2 active form (Figure 3C).

### 2.5. TRPML2 Silencing Triggers the Migration/Invasion in T98 and U251 Cells

**Figure 4:**
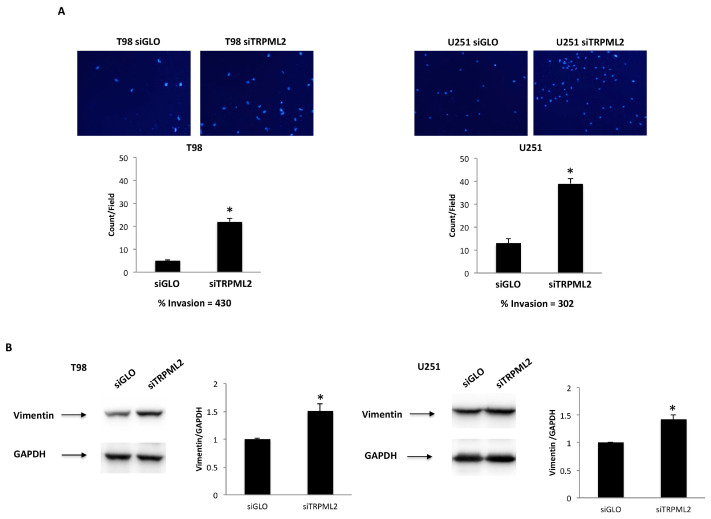
TRPML2 silencing increases the invasion capability in T98 and U251 cell lines. (**A**) Representative images showing DAPI fluorescence after 24 h culture in Transwell chambers (×10 magnification). The siGLO and siTRPML2 T98 and U251 invading cells were counted in 10 randomly chosen microscopic fields per transwell. Each sample was run in triplicate, and three independent experiments were performed. Bars represent the quantification of invaded cells in each field (Mean ± SE), * *p* < 0.01. (**B**) Representative immunoblots reflecting vimentin protein levels in siGLO and siTRPML2 T98 and U251 cell lines. Blots are representative of one of three separate experiments. Densitometry values were normalized to GAPDH, which was used as a loading control. Data are expressed as the mean ± SE of three separate experiments. * *p* < 0.05.

Then, we evaluated the invasion capability of both siGLO and siTRPML2 T98 and U251 cells. The transwell invasion assay was performed in 24-well plates precoated with Matrigel. A marked invasion capability was observed in both siTRPML2 cells (Figure 4A) compared with siGLO T98 and U251, respectively. Moreover, the effects of TRPML2 activation or of enforced TRPML2 expression on invasion were also evaluated. No changes in the invasion capability were observed in ML2-SA1-stimulated or TRPML2 overexpressing T98 and U251 cells, compared with respective controls (Figures S4 and S5).

The VEGFA reduction and the increased expression of EMT markers (e.g., vimentin and CD44) promote the invasion capability [37]. Thus, we further evaluated whether the pro-invasive phenotypes we observed in invasive siTRPML2 cells, associated with reduced VEGFA and increased vimentin, mRNA expression (Table 1 and Table 2), were also accompanied by increased vimentin protein levels. By Western blot, we found vimentin protein upregulation in both siTRPML2 cells, compared with control cells (Figure 4B). 

### 2.6. TRPML2 Silencing or ML2-SA1 Treatment Modulates the Doxorubicin Resistance in T98 and U251 Cells

**Figure 5:**
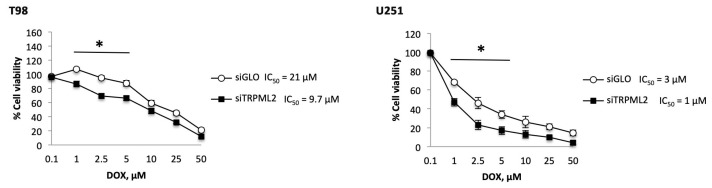
TRPML2 silencing affects the DOX resistance in T98 and U251 cell lines. Cell cytotoxicity was evaluated by 3-(4,5-dimethylthiazol-2-yl)-2,5-diphenyltetrazolium bromide (MTT) assay in siGLO and in siTRPML2 T98 and U251 cell lines, treated with DOX at different doses for 24 h. Data shown are expressed as the mean ± SE of three separate experiments. * *p* < 0.05 siTRPML2 vs. siGLO.

The mesenchymal transition in GBM is associated with the acquisition of an aggressive phenotype and drug resistance [38,39,40]. Thus, the effect of TRPML2 silencing or TRPML2 activation using different doses of ML2-SA1 agonist for 24 h was evaluated in GBM cells treated with Doxorubicin (DOX: 1–50 μM). Silencing of TRPML2 mRNA in T98 and U251 increases the sensitivity of glioma cells to the DOX cytotoxic effects (siGLO vs. siTRPML2 T98 cells, IC_50_ = 21 vs. 9.7 μM; siGLO vs. siTRPML2 U251 cells, IC_50_ = 3.0 vs. 1.0 μM), suggesting that TRPML2 can contribute to DOX resistance in GBM cells (Figure 5).

**Figure 6:**
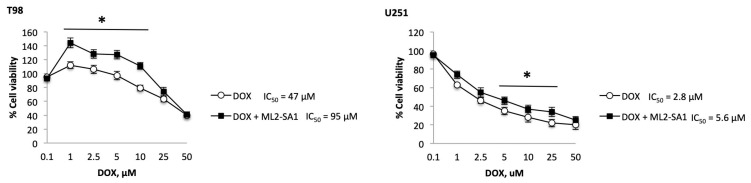
ML2-SA1 treatment affects the DOX (Doxorubicin)-resistance in T98 and U251 cell lines. Cell cytotoxicity was evaluated by 3-(4,5-dimethylthiazol-2-yl)-2,5-diphenyltetrazolium bromide (MTT) assay in glioma cell lines, treated with DOX at different doses for 24 h after the pretreatment with ML2-SA1 for 6 h. Data shown are expressed as the mean ± SE of three separate experiments. * *p* < 0.05.

In addition, treatment of T98 and U251 cells with the TRPML2 agonist, ML2-SA1, at 10 μM, increased the resistance to DOX-induced cytotoxic effects in both cell lines (vehicle- vs. ML2-SA1-treated T98 cells, IC50 = 47.0 vs. 95.0 μM; vehicle- vs. ML2-SA1-treated U251 cells, IC50 = 3.5μM vs. 5.6 μM) (Figure 6).

### 2.7. Silencing of TRPML2 Triggers pRB Degradation in T98 and U251 Cells

RB is a tumor suppressing gene regulating survival, proliferation and differentiation in GBMs; moreover, a role of pRB1 in lysosome acidification, vesicle trafficking and autophagosome/lysosome fusion process has also been identified [41,42]. 

**Figure 7:**
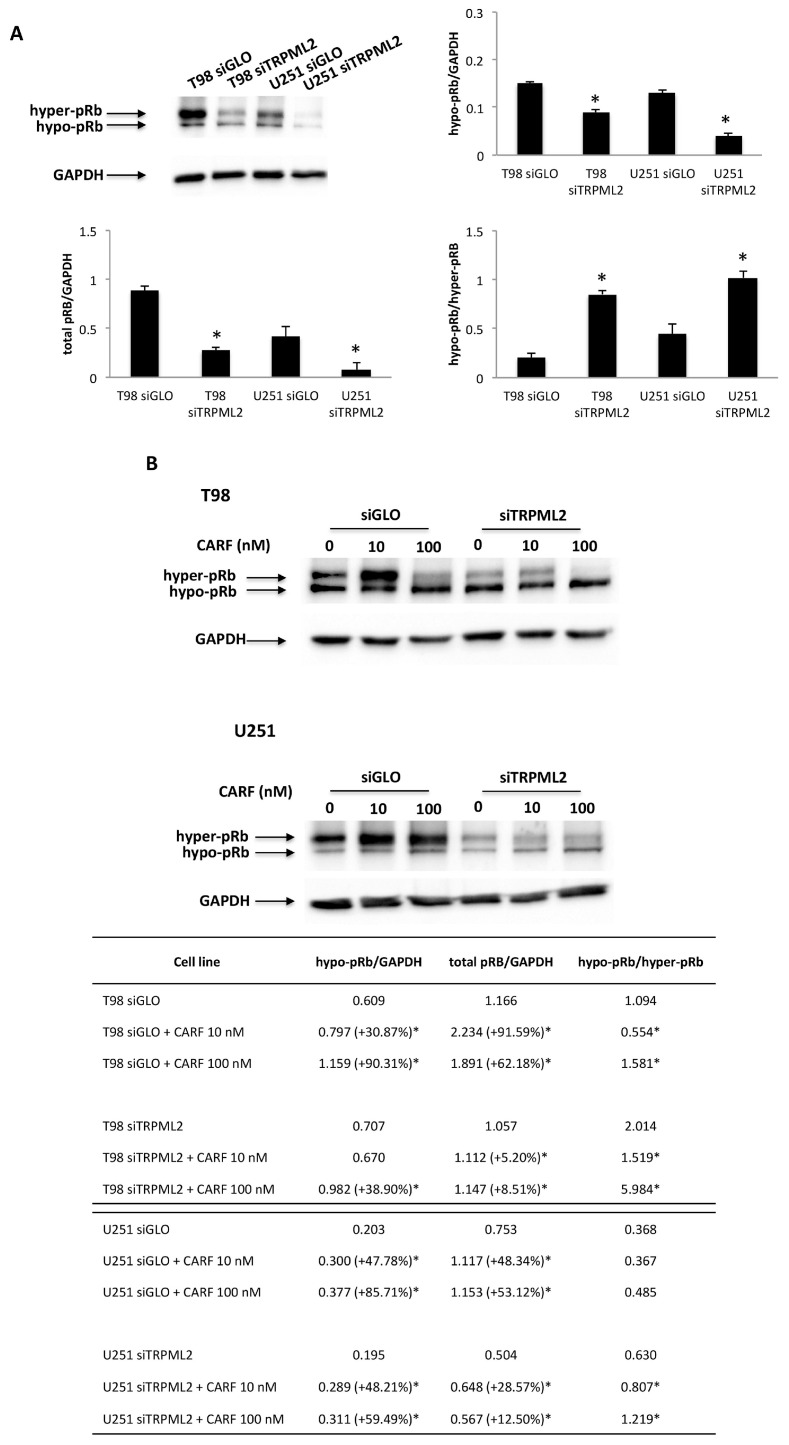
TRPML2 silencing reduces pRB1 levels and the proteasome inhibitor Carfilzomib partially reverts this inhibitory effect in T98 and U251 cell lines. (**A**) Representative immunoblot regarding pRB1 in siGLO and siTRPML2 T98 and U251 cell lines 48 h post-transfection. Blots are representative of one of three separate experiments. Densitometry values were normalized to GAPDH, which was used as a loading control. Data are expressed as themean ± SE of three separate experiments. * *p* < 0.05 siTRPML2 vs. siGLO. (**B**) Representative immunoblot regarding pRB1 in CARF (Carfilzomib)-treated siGLO and siTRPML2 T98 and U251 cells. Blots are representative of one of three separate experiments. Densitometry values were normalized to GAPDH, which was used as a loading control. Data are expressed as the mean ± SE of three separate experiments. * *p* < 0.01 CARF vs. vehicle.

Herein, the effects of TRPML2 silencing in T98 and U251 cells on the pRB expression and activation were evaluated in siTRPML2 and siGLO T98 and U251 cells. We found that TRPML2 knock-down in glioma cells reduces total pRB1 as well as the active/hypophosphorylated form levels, compared with siGLO T98 and U251 cells. Additionally, the hypo/hyperphosphorylated pRB protein ratio was increased (Figure 7A).

Then, the involvement of the proteasome in TRPML2-dependent pRB degradation was evaluated. T98 and U251 cells were treated with the proteasome inhibitor, Carfilzomib (CARF), at 10 nM and 100 nM for 24 h. Results demonstrated that 10 nM CARF increases the total pRB levels (91% and 53% in T98 and U251 cells, respectively) (Figure 7B). Pretreatment of GBM cells with 10 nM of CARF, partially reverted, the TRPML2-mediated pRB reduction, by increasing the hypophosphorylated-pRb form and total pRB levels as well as the hypo/hyperphosphorylated-pRb ratio. These results suggest a contribution of proteosome in the TRPML2-induced pRB degradation. Moreover, strong increases in hypo-pRb and parallel decreases in hyper-pRb forms were evidenced in 100 nM CARF-treated T98 cells compared with U251 cells (Figure 7B). 

**Figure 8:**
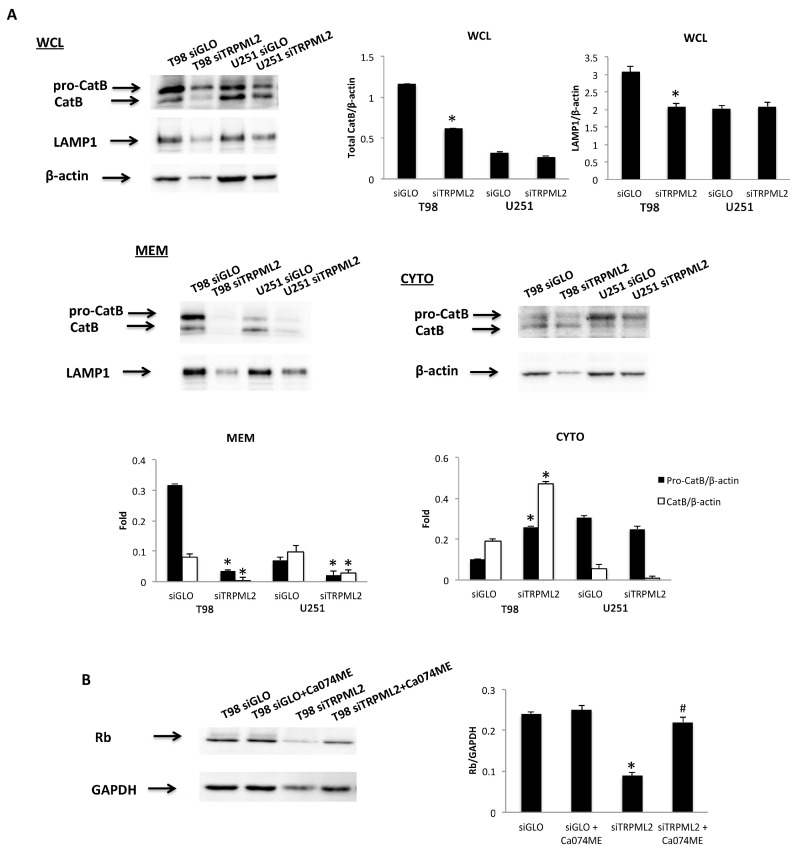
Influence of TRPML2 silencing on Cathepsin B. (**A**) Proteins derived from the cytosolic fraction (Cyto), membrane fraction (MEM) and whole cell lysate (WCL) were immunoblotted with anti-Cathepsin B (CatB) Ab. The purity of subcellular fractions was assessed by blotting Cyto and WCL against β-actin, and MEM and WCL against LAMP1. Blots are representative of one of three separate experiments. Data are expressed as the mean densitometric values ± SE of three separate experiments. * *p* < 0.05 siTRPML2 vs. siGLO. (**B**) Representative Western blot analysis of pRb in Ca074ME-treated siGLO and siTRPML2 T98 cells. Blots are representative of one of three separate experiments. Densitometry values were normalized to GAPDH, which was used as loading control. Data are expressed as the mean ± SE of three separate experiments. * *p* < 0.01 siTRPML2 vs. siGLO and siGLO + Ca074ME, ^#^ *p* < 0.01 siTRPML2 + Ca074ME vs. siTRPML2.

A role of the knock-out of TRPML channels in Cathepsin B (CatB) maturation and release [43] as well as the sensitivity of pRB to CatB-like degradation [44] was demonstrated; therefore, the CatB involvement in TRPML2-mediated effects has been evaluated. CatB localization in siTRPML2 and siGLO T98 and U251 cells was established using a subcellular fractionation protocol. Immunoblots showed that although the pro-CatB and the mature CatB protein levels were reduced in total and membrane fractions in both siTRPML2 T98 and U251 cells, compared with siGLO cells, the cytosolic CatB levels in T98, but not U251 siTRPML2 cells, were significantly increased, compared with siGLO T98 cells (Figure 8A). 

Finally, to further sustain the contribution of CatB in siTRPML2-mediated pRB degradation in T98 cells, siTRPML2 and siGLO cells were pretreated in the last 24 h of the 72 h of transfection with the intracellular CatB inhibitor, CA074ME [L-3-trans-(Propylcarbamoyl)oxirane-2-carbonyl]-L-proline methyl ester, (5 μM) [45]. We found that the siTRPML2-mediated pRB reduction is markedly reverted in Ca074ME-treated siTRPML2, compared with siGLO T98 cells (Figure 8B). No major difference was observed when comparing siGLO or siGLO plus Ca074ME T98 cells.

### 2.8. TRPML2 Overexpression as a Negative Prognostic Factor in GBM Patients

**Figure 9:**
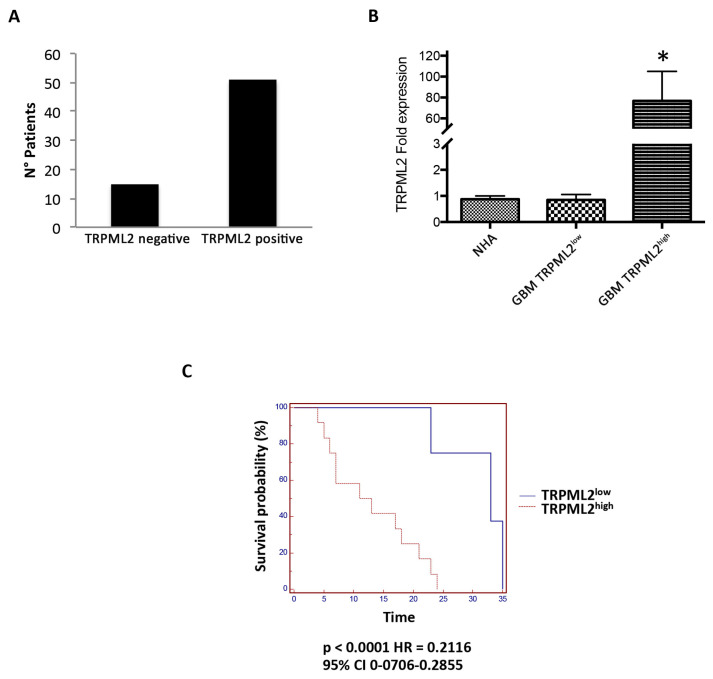
TRPML2 overexpression correlates with poor prognosis in GBM patients. (**A**) Number of patients TRPML2 negative and TRPML2 positive. (**B**) The relative TRPML2 mRNA expression in normal human astrocyte (NHA), and GBM samples expressing low (n = 18) or high (n = 33) TRPML2 was evaluated by qRT-PCR. TRPML2 mRNA levels were normalized for GAPDH expression. Data are expressed as the mean ± SD. * *p* < 0.01 vs. NHA and TRPML2 low samples. (**C**) Kaplan–Meier plot for GBM patients in TRPML2^low^ and TRPML2^high^ subgroups. *p* < 0.0001.

The expression of TRPML2 was evaluated at mRNA level in human GBM tissues (n = 66) (Table S1) and NHA cell lines (n = 2). Then, we calculated the mean and the median OS of GBM patients. We found that the mean OS was 14.4 months and the median OS was 11.0 months. About 77.3% (n = 51/66) of GBM tissues expressed TRPML2 mRNA, whereas 22.7% (n = 15/66) of the samples were TRPML2 negative (Figure 9A). Then, we sub-grouped the TRPML2 positive mRNA samples (n = 51/66) in TRPML2^high^ (n = 33/51) and TRPML2^low^ (n = 18/51), as evaluated by ROC analysis and compared them with NHA. Strong significant differences were evidenced between TRPML2^high^ and TRPML2^low^ or NHA (Figure 9B). Through Kaplan–Meier analysis, we evaluated the correlation between patients’ OS and TRPML2 mRNA expression. The median OS of TRPML2^high^ patients was significantly shorter than that of TRPML2^low^ (11 months vs. 33 months; *p* < 0.0001) (Figure 9C). Concordantly, through univariate analysis, a statistically significant difference in OS was evidenced between TRPML2^high^ and TRPML2^low^ GBM patients (*p* < 0.0001, 95% CI 3.8305–41.4779).

In conclusion, high TRPML2 levels strongly correlate with short survival in GBM patients, supporting TRPML2 as a negative independent prognostic factor in GBM patients.

## 3. Discussion

A key feature of tumor growth and development is angiogenesis. Robust vascularization, often associated with the overexpression of VEGFA, is a hallmark of GBM [22]. VEGFR2 (KDR, Flk-1) is the major pro-angiogenetic receptor for VEGFA-induced signaling in endothelial cells. Herein, we found that the silencing of TRPML2 reduces the VEGFA mRNA and protein levels, VEGFA protein release and VEGFR2 protein expression, compared with control cells. TRPML2 activation through ML2-SA1 enhances the VEGFA and VEGFR2 protein expression and increases the VEGFA protein release. In addition, enforced pCMV-TRPML2 expression increases the VEGFR2 protein expression and VEGFA protein release in pTRPML2 compared with pCMV T98 and U251 cells. 

Signaling from VEGFR2 is necessary for VEGF-stimulated proliferation, chemotaxis, sprouting and angiogenesis in vivo [46,47,48]. Upon ligand binding, VEGFR2 undergoes autophosphorylation and becomes activated [49], leading to the recruitment of Shc, GRB2, PI3 kinase, NCK, SHP-1 and SHP-2 [50]. Major autophosphorylation sites of VEGFR2 are in the kinase insert domain (TyR951/996), the tyrosine kinase catalytic domain (TyR1054/1059) [51] and TyR1175, which provide a docking site for the p85 subunit of PI3 kinase, PLCγ and Shb [40,52,53]. Specific functions of TyR996 phosphorylation were not clear. Herein, we found that VEGFR2 was phosphorylated at basal level at the 996TyR and 1175TyR sites in siGLO T98 and U251 cells. Silencing of TRPML2 inhibits the VEGFR2-Tyr996 and VEGFR2-TyR1175 phosphorylation in both siTRPML2 GBM cells. The roles of TyR1175 in ERK-dependent proliferation, Akt activation and migration [40] and inhibition of TyR1175 VEGFR2 we observed in siTRPML2 T98 and U251 cells are in agreement with our previously reported data on the inhibition of ERK-dependent proliferation and AKT activation in siTRPML2 GBM cell lines [2]. However, treatment of T98 and U251 cells with the ML2-SA1 agonist or enforced TRPML2 expression does not induce changes inVEGFR2 tyrosine phosphorylation. 

The Notch signaling is important in the proliferation, differentiation, apoptosis, and regulation of GBM functions [54]. Moreover, it is also involved in hypoxia and angiogenesis responses, which are typical GBM features [29,30,31]. Notch receptors are activated in gliomas, and their oncogenicity has been confirmed by gain- and loss of-function studies in vitro and in vivo [28]. In our models, Notch2 signaling was activated at basal levels, as evidenced by the presence of NICD Notch2 proteolytic fragment in both GBM cell lines. Silencing or enforcement of TRPML2 expression reduced or enhanced Notch2 protein activation levels, respectively. Finally, the treatment of T98 and U251 cells with different doses (10 and 30 μg/mL) of the ML2-SA1 agonist increased Notch2 NICD in ML2-SA1 as compared with siGLO vehicle-treated cells. The reduction in Notch2 NICD in siTRPML2 T98 and U251could be the result of impaired endolysosome function and V-ATPase-dependent acidification, induced by TRPML2 deregulation, required for activating Notch enzymatic cleavage [55]. In contrast, the increase in Notch2 NICD levels in pCMV-TRPML2 compared with pCMV T98 and U251 cells may be due to an increased protease activity causing Notch2 protein degradation. 

The EMT process in GBM is associated with the acquisition of drug-resistance and pro-invasive features [40]. In GBM patients, the use of Anthracyclines, such as DOX, which induce apoptotic/necrotic cell death in vitro and in vivo could represent an alternative to TMZ-based chemotherapy; however, due to their exclusion by the blood-brain barrier [56,57], only recently, thanks to the application of a number of new vehicle delivery strategies [58], it is now possible to treat GBM tumors with DOX. In this regard, we have demonstrated that the silencing of TRPML2 mRNA in T98 and U251 results in the increase in the DOX sensitivity (IC50: from 9.7 to 21.0 and from 1.0 to 3.0 μM in T98 and U251, respectively), suggesting that high levels of the TRPML2 channel contributes to DOX resistance in GBM cells. Moreover, in contrast, treatment of GBM cell lines with the ML2-SA1 agonist impaired DOX sensitivity (IC50: from 47.0 to 95.0 and 3.5 to 5.6 μM, respectively). 

We also demonstrated that the silencing of TRPML2 increases the migration/invasion capability in T98 and U251 cells compared with siGLO cells. Our data, in the view of the role of TRPML2 in the triggering chemokine trafficking and secretion in murine macrophages [15,59], seems to be contradictory. However, the pro-invasive phenotype observed in siTRPML2, compared with siGLO cells, may be a result of VEGFA/VEGFR2 pathway downregulation, which stimulates the EMT process in GBM cell lines, resulting in the acquisition of a more invasive mesenchymal phenotype [38,39]. Furthermore, in recent years it has been demonstrated that in astrocytoma cells, proliferation and migration are timely separated, and this phenomenon is called “Go or Grow” [60]. Indeed, the behavior of cancer cells, modulated by changes in the microenvironment such as hypoxia, nutrient depletion and extracellular matrix composition, switches between “Go”, when cells migrate in order to reach a more favorable environment, or “Grow”. Ion channels seem to have a significant role in this process and TRPML2 could be an actor, given that its silencing decreases cell proliferation [2] and induces an invasive mesenchymal-like phenotype. Indeed, increases in the EMT marker, vimentin, both at mRNA and protein levels were demonstrated in siTRPML2 cell lines. In this regard, in a mouse model of GBM, the VEGFA blockade was observed and in GBM patients treated with bevacizumab, a pro-invasive phenotype with increased cell migration/invasion, associated with a mesenchymal phenotype with high vimentin levels was reported [61]. Moreover, accordingly with a pro-invasive behavior, increased SPARC mRNA levels were evidenced by dd-PCR in siTRPML2 compared with siGLO T98 and U251 cells. SPARC is highly expressed in GBM, where it promotes the migratory and invasive behavior of glioma cells [62]; moreover, by suppressing tumor vascularity, through the abrogation of VEGFA expression and inhibition of VEGFR2 phosphorylation, it inhibited glioma growth and VEGF-induced DNA synthesis [63]. In contrast, the stimulation of T98 and U251 cells with different doses of ML2-SA1 agonist for 24 h or enforcing TRPML2 expression does not influence the invasion capability in T98 and U251 cells. These data are in agreement with previously reported data in murine macrophages in the inability of ML2-SA1 to stimulate migration in the absence of a second signal. In fact, ML2-SA1 only stimulates direct CCL2 release and subsequent macrophage migration in LPS-activated macrophages [15,59]. 

Lysosomes are cellular organelles involved in degradation and recycling processes. Luminal pH is critical in these processes because the lysosomal hydrolases only function optimally in the acidified lysosomal microenvironment [64]. The RB1 is a tumor-suppressing gene regulating survival, proliferation, angiogenesis, migration, differentiation, apoptosis, senescence and drug-resistance [32]. Huang et al. have shown that the cleavage of pRB1 by caspase or with bafilomycin 1, an inhibitor of the lysosomal H+ condensation through the vacuolar type H(þ)-ATPase, inactivates pRB1 [41]. Here, we found that TRPML2 silencing in GBM cells reduces the total pRB1 and the active/hypophosphorylated pRB1 protein levels, compared with siGLO T98 and U251 cells. Then, the contribution of the proteasome in the TRPML2-dependent degradation of pRB1 protein in glioma cell lines was evaluated using CARF, a selective proteasome inhibitor [65]. Pretreatment of siTRPML2 T98 and U251 cells for 24 h with different doses (10 and 100 nM) of CARF increases total pRB1 and hypo-phosphorylated pRB1 levels in siGLO T98 and U251 cells. Moreover, in siTRPML2 cells CARF partially, but not completely, reverted the TRPML2-mediated reduction in pRB1 levels, by increasing the hypophosphorylated pRB1 form in siTRPML2, compared with siGLO T98 and U251 cells. 

The pRB protein shows high susceptibility to cytosolic proteases, with CatB-like proteolytic activity [44]; moreover, we and other researchers [43,66], have previously reported that TRPML1 knock-down results in the leak of mature lysosomal CatB. In this regard, increased levels of mature CatB were evidenced in the cytosol fraction of siTRPML2 T98 cells, but not U251 cells compared with siGLO control cells. Moreover, pretreatment of siTRPML2 T98 cells for 24 h with the CatB inhibitor, Ca074ME, markedly reverts the siTRPML2-mediated reduction in pRB protein levels, compared with siGLO T98 cells. Overall, these data suggest that in TRPML2 silenced T98 cells, proteasome and CatB proteolytic activity combine to reduce the pRB1 protein levels; other proteases other than proteasome activity are likely required in siTRPML2 U251 cells. 

Taken together, because of the pleiotropic effects of pRB in angiogenesis, migration, drug-resistance as well as lysosome functions in GBM cells, we can hypothesize that the siTRPML2-mediated reduction in pRB1 protein expression, can also contribute to VEGFA/VEGFR2 inhibition, Notch 2 activation, increased migration and DOX-resistance reported inTRPML2-silenced GBM cell lines. 

Finally, the correlation between the survival of GBM patients and the TRPML2 mRNA expression was studied. Through RT-PCR analysis, we found that 15/66 GBM patients were negative for TRPML2 expression. Through ROC analysis we categorized the 51/66 TRPML2-positive GBM patients into TRPML2^high^ (37/51 samples) and TRPML2^low^ (14/51 samples) mRNA expression. Through Kaplan-Meier survival analysis, we found that high TRPML2 expression was strongly correlated with short OS (11 months), whereas low TRPML2 expression was associated with a more favorable OS (33 months) in GBM patients, suggesting that TRPML2 mRNA overexpression represents a negative prognostic factor in GBM patients. Similar results were obtained with univariate COX hazard analysis, with high TRPML2 expression maintaining an independent negative prognostic significance for the survival in GBM patients. 

Overall, in GBM patients TRPML2 mucolipin channels seem to play an important role in the regulation of the VEGFA/VEGFR2/NOTCH2 signaling pathway as well as in invasion, DOX-resistance and CatB-dependent and -independent pRB1 proteasomal degradation. Moreover, the OS of GBM patients is controlled by the fine balance between both survival/proliferation and migration/invasion, and TRPML2 seems to participate in this dynamic control. Enhanced TRPML2 expression sends a “Growth” signal that promotes survival, angiogenesis and proliferation; in contrast, the loss/downregulation of TRPML2 drives a “Go” signal triggering the EMT and migration/invasion processes. 

Therefore, understanding the biology governing this equilibrium in GBM is of great importance for developing new therapeutic approaches to treat GBM patients.

## 4. Materials and Methods

### 4.1. Cells and Tissues

Formalin-fixed paraffin-embedded brain tissues from human tumor biopsies and epileptic brain (EHB) (n = 2) surgically removed from patients who gave informed consent to the study (n = 66), were kindly provided by Prof. Arcella Antonella (I.N.M., Neuromed, Pozzilli, Isernia, Italy). Glioblastoma tissues (grade IV) were histologically graded according to the World Health Organization classification criteria. Patients were eligible for the study if a diagnosis of glioblastoma was established histologically according to the WHO classification [67]. Informed consent was obtained before surgery according to the Neuromed Ethics Committee The glioblastoma T98 and U251 cell lines (grade IV), obtained from European Collection of Cell Cultures (ECACC, Salisbury, UK), were maintained in Eagle’s minimum essential medium (EMEM, Lonza Bioresearch, Basel, Switzerland) supplemented with 10% heat-inactivated fetal bovine serum (FBS), 2 mmol/L L-glutamine, 100 IU/mL penicillin, and 100 μg streptomycin. 

### 4.2. Chemical and Reagents

ML2-SA1 (EVP-22) was purchased from Axon Medchem BV (Groningen, The Netherlands). The 3-(4,5-dimethylthiazol-2-yl)-2,5-diphenyltetrazolium bromide (MTT), doxorubicin (DOX), and carfilzomib (CARF) were purchased from Sigma-Aldrich (Milan, Italy). 4′,6-diamidino-2-phenylindole (DAPI) was purchased from BioRad (Milan, Italy).

The following antibodies (Abs) were used: mouse anti-TRPML2 (1:300, Santa Cruz Biotechnology, Dallas, TX, USA), mouse anti-pRB1 (1:300, Santa Cruz Biotechnology, Dallas, TX, USA), rabbit anti-Notch2 (1:1000, Cell Signaling Technology, Danvers, MA, USA), mouse anti-VEGFA (1:300, Santa Cruz Biotechnology, Dallas, TX, USA), rabbit anti-VEGFR2 (1:1000, Cell Signaling Technology, Danvers, MA, USA), rabbit anti-VEGFR2(Tyr951) (1:1000, Cell Signaling Technology, Danvers, MA, USA), rabbit anti-VEGFR2 (Tyr996) (1:1000, Cell Signaling Technology, Danvers, MA, USA), rabbit anti-VEGFR2 (Tyr1059) (1:1000, Cell Signaling Technology, Danvers, MA, USA) and rabbit anti-VEGFR2 (TyR1175) (1:1000, Cell Signaling Technology, Danvers, MA, USA), mouse anti-LAMP1 (1:300, Santa Cruz Biotechnology, Dallas, TX, USA), mouse anti-glyceraldehyde-3-phosphate dehydrogenase (anti-GAPDH, 1:1000, Santa Cruz Biotechnology, Dallas, TX, USA) and mouse anti-βactin (1:1000, Santa Cruz Biotechnology, Dallas, TX, USA).The following secondary antibodies were used: horseradish peroxidase (HRP)-conjugated anti-mouse IgG and HRP-conjugated anti-rabbit IgG (1:2000, Cell Signaling Technology, Danvers, MA, USA). 

### 4.3. MTT Assay

For the MTT assay, 3 × 10^4^/mL cells were plated in 96-well plates and treated with different doses of ML2-SA1 (1–50 μM) and DOX (1–50 μM), alone or in combination. Then, 0.8 mg/mL of MTT was added to the samples and incubated for additional 3 h. After the removal of medium from the wells, the formazan crystals were dissolved with 100 µL per well of DMSO and the colored solutions were read by microtiter plate spectrophotometer (BioTek Instruments, Winooski, VT, USA). Four replicates were used for each treatment. The drug concentration that induced 50% cell growth inhibition compared with control cells (IC50) was calculated using GraphPad Prism^®^ 5.0a (GraphPad Software, San Diego, CA, USA). 

### 4.4. Immuno-Quantitative Enzyme-Linked Immunosorbent Assay (ELISA) for VEGFA

For ELISA, 4 × 10^4^/mL cells were plated in 24-well plates. Conditioned media were collected 24 h later and subjected to RayBio Human VEGF IQELISA Kit (RayBiotech, Peachtree Corners, GA, USA) to measure the levels of VEGFA protein following the manufacturer’s instructions.

### 4.5. Western Blot Analysis

To obtain whole cell lysates, cells were lysed in a lysis-buffer containing protease inhibitor cocktail (EuroClone, Milan, Italy). Cytoplasmatic and membrane/organelles were isolated using the Cell Fractionation Kit (Cell Signaling Technology, Danvers, MA, USA) according to the manufacturer’s instruction. Proteins were separated on SDS polyacrylamide gel in a Mini-PROTEAN Tetra Cell system (BioRad, Milan, Italy) and transferred to a nitrocellulose membrane using Mini Trans-Blot Turbo RTA system (BioRad, Milan, Italy). Non-specific binding sites were blocked with 5% low-fat dry milk or 5% bovine serum albumin (BSA) in phosphate-buffered saline 0.1% Tween 20 for 1 h at room temperature. Membranes were incubated overnight at 4 °C in primary Abs (anti-TRPML2, anti-VEGFA, anti-VEGFR2, anti-TyR959, anti-TyR996, anti-TyR1059 and anti-TyR1175, anti-NOTCH2, anti-LAMP1, anti-β-actin and anti-GAPDH), followed by the incubation for 1 h at room temperature with HRP-conjugated anti-rabbit or anti-mouse secondary Abs. The detection was performed using the LiteAblot PLUS or Turbo kits (EuroClone, Milan, Italy), and densitometric analysis was carried out by a Chemidoc using the Quantity One software (version 4.6.7, BioRad, Milan, Italy). For quantification, GAPDH and β-actin were used as loading control. One representative out of three independent experiments is shown in each immunoblot. 

### 4.6. Invasion Assay

Cell invasion was evaluated by Transwell assay using the Transwell Chambers (BD Biosciences, Franklin Lakes, NJ, USA) as previously described [65]. Briefly, a total of 750 μL cell culture medium supplemented with 10% FBS was added in the lower chamber. A serum-free culture medium (500 μL) containing 2.5 × 10^4^ glioma cells was plated into the upper chamber. After 24 h of incubation, cells remaining in the upper chamber were removed by cotton swabs. The cells were fixed in 4% paraformaldehyde and stained with DAPI. Fluorescence microscope (BX51 Fluorescence Microscope, Olympus, Milan, Italy) and Image J software version 1.45 s (National Institutes of Health, Bethesda, MD, USA) were used for the images acquisition at ×10 magnification and analysis. Ten fields were selected at random to measure the average cell coverage. The experiments were performed in triplicate at least three times independently.

### 4.7. TRPML2 Transfection Models

For silencing experiments, TRPML2 (siTRPML2) and siCONTROL non-targeting siRNA (siGLO, used as negative control) FlexiTube siRNA were purchased from Qiagen (Milan, Italy). For gene silencing experiments, T98 and U251 cell lines were plated at a density of 1.2 × 10^5^/mL and siTRPML2 or siGLO (150 ng) was added to the wells, following the HiPerfect transfection reagent transfection protocol (Qiagen, Milan, Italy). No differences were detected comparing siGLO control cells with untransfected cells. 

For overexpression experiments, 1.5 × 10^5^/mL T98 and U251 cells were plated. After overnight incubation, transfections were achieved with 10 µL/well of Roti-Fect (Carl Roth GmbH, Karlsruhe, Germany) and 2 µg/well of pCMV3-*MCOLN2*-t1 (pCMV-TRPML2) (Sino Biological, Wayne, PA, USA) or pCMV3 empty (pCMV) vectors according to the manufacturer’s instructions. No differences were observed comparing pCMV transfected with untransfected cells.

### 4.8. Gene Expression Analysis

Total RNA from fixed paraffin-embedded tissue slices (5–7 μm thick) was extracted by RNeasy^®^ FFPE Mini Kit (Qiagen, Milan, Italy) and cDNA was synthesized using the High-Capacity cDNA Archive Kit (Applied Biosystems, Foster City, PA, USA) according to the manufacturer’s instructions. Then, 5 μL of the cDNA was pre-amplified for 15 cycles using SsoAdvancedPreAmp Supermix kit (BioRad, Milan, Italy). One microliter of the resulting cDNA products was used as template for quantitative real-time polymerase chain reaction (qRT-PCR).

Quantitative RT-PCR was performed by using the IQ5 Multicolor real-time PCR detection system (BioRad, Milan, Italy). The reaction mixture contained the Advanced Universal SYBRGreen Supermix (BioRad, Milan, Italy). Human TRPML2 and GAPDH RT^2^ qPCR Primer assay (Qiagen, Milan, Italy) were used. The PCR parameters were 10 min at 9 °C, and 40 cycles at 9 °C for 15 s and 60 °C for 40 s. All samples were assayed in triplicate in the same plate. The relative amount of target mRNA was calculated by the 2^−ΔΔCt^ method. GAPDH was used as a housekeeping gene.

RNA from U251 and T98 cell lines was extracted by Single Shot Cell Lysis Kit (BioRad, Milan, Italy) according to the protocol. Subsequently, 800 ng of extracted RNA was subjected to reverse transcription in a total volume of 20 μL using the iScript kit (BioRad, Milan, Italy) according to the manufacturer’s instruction sand the resulting cDNA was used to preamplify each sample for all primers used in the gene expression analysis by SSOADvancedPreAmp Kit and Assays (BioRad, Milan, Italy). The ddPCRSupermix for Probes (No dUTP) (BioRad, Milan, Italy) and the specific Prime PCR^TM^ ddPCR^TM^ Expression Probe Assays conjugated with FAM or HEX fluorescent dyes (the same pool used in the pre-amplification step) (BioRad, Milan, Italy) were then used to perform the digital droplet-PCR (ddPCR). The analyzed target genes were: VEGFA, NOTCH1/2, VEGFB, EPCAM, SPARC, STAT3, VIMENTIN, CD44, SHH, DHH, IHH, PTCH1/2, ZEB1/2 and SMO. Results, expressed as cDNA copies/μL were normalized to β-actin concentration and analyzed using the QuantaSoft Software (BioRad, Milan, Italy). 

### 4.9. Statistical Analysis

The statistical significance was determined by Student’s t-test and by ANOVA with Bonferroni’s post-test. Overall survival was defined as the interval between the date of surgery to death or last follow-up visit. Median overall survival (OS) was estimated using Kaplan–Meier method with Rothman’s 95% confidence intervals (CI) and compared across the groups using the log-rank test. For univariate analysis of significance, the long-rank test or Cox analysis was used. Regarding TRPML2 mRNA expression GBM patients (n = 66) were 15/66 negative and 51/66 positive. These positive patients, subgrouped into TRPML2 high and low, were subjected to survival analysis. Kaplan–Meier analysis was performed stratifying patients in TRPML2^low^ < 6.3 and TRPML2^high^ > 6.3, according to relative operating characteristic (ROC) analysis. Statistical analysis was performed with MedCalc package (MedCalc^®^ version 16.4.3, Ostend, Belgium). 

## Figures and Tables

**Table 1:** Effects of TRPML2 silencing in the T98 cell line.

Target Gene	T98 siGLO	T98 siTRPML2
ALCAM	826 ± 12	854 ± 9
CD44	4510 ± 17	7800 ± 23 *
VIMENTIN	6421 ± 19	7270 ± 7 *
ZEB1	566 ± 8	540 ± 12
ZEB2	44 ± 2	50 ± 3
VEGFA	1506 ± 4	1273 ± 5 *
VEGFB	1492 ± 7	1634 ± 10 *
NOTCH1	34 ± 4	30 ± 2
NOTCH2	1492 ± 12	988 ± 9 *
STAT3	1090 ± 14	1368 ± 17 *
SPARC	1835 ± 15	2810 ± 15 *
EPCAM	5 ± 0	3 ± 0
SHH	0 ± 0	0 ± 0
DHH	7 ± 1	0 ± 0
IHH	0 ± 0	0 ± 0
PTCH1	83 ± 3	79 ± 2
PTCH2	2 ± 0	2 ± 0
SMO	257 ± 3	246 ± 6
POU5F1B	0 ± 0	0 ± 0
ACTB	7400 ± 37	7500 ± 53

Data are expressed as the mean (cDNA copies/μL) + SD of two separate experiments, normalized for the ACTB housekeeping gene. Abbreviations: ALCAM/CD166: activated leucocyte cell adhesion molecule; CD44: CD44; EPCAM: epithelial cell adhesion molecule; SHH: sonic hegdehog; DHH: desert hedgehog; IHH: indian hedgehog; PTCH1: patched 1; PITCH2: patched 2; ZEB1: zinc finger E-box binding homebox 1; ZEB2: zinc finger E-box binding homebox 2; SMO: smoothened frizzled class receptor; VEGFA, vascular endothelial growth factor A; VEGFB, vascular endothelial growth factor B; NOTCH1: notch receptor 1; NOTCH2: notch receptor 2; POU5F1B: POU class 5 homebox 1B; STAT3: signal transducer and activator of transcription 3; SPARC: secreted protein acidic and cystein rich; ACTB: β-actin. * *p* < 0.05 vs. T98 siGLO.

**Table 2:** Effects of TRPML2 silencing in the U251 cell line.

Target Gene	U251 siGLO	U251 siTRPML2
ALCAM	1153 ± 13	1218 ± 9
CD44	10,200 ± 21	10,300 ± 16
VIMENTIN	6740 ± 12	7900 ± 10 *
ZEB1	110 ± 3	143 ± 6
ZEB2	167 ± 5	130 ± 4
VEGFA	3440 ± 16	1617 ± 13 *
VEGFB	537 ± 7	698 ± 10 *
NOTCH1	381 ± 7	132 ± 6 *
NOTCH2	2020 ± 12	1476 ± 14 *
STAT3	984 ± 13	1192 ± 6 *
SPARC	1964 ± 9	2254 ± 6 *
EPCAM	0 ± 0	2 ± 0
SHH	5 ± 0	1 ± 0
DHH	3 ± 1	1 ± 0
IHH	4 ± 0	1 ± 0
PTCH1	27 ± 2	32 ± 1
PTCH2	102 ± 3	59 ± 3
SMO	37 ± 2	54 ± 2
POU5F1B	1 ± 0	2 ± 0
ACTB	11,300 ± 32	11,300 ± 46

Data are expressed as the mean (cDNA copies/μL) + SD of two separate experiments, normalized for the ACTB housekeeping gene. Abbreviations are as described in Table 1. * *p* < 0.05 vs. U251 siGLO.

## Data Availability

The data that support the findings of this study are available from the corresponding authors upon request.

## Supplementary Materials

The following supporting information can be downloaded at: https://www.mdpi.com/article/10.3390/ijms23020688/s1.
