# Supplementary figures and images for: Functional In Vitro Assessment of VEGFA/NOTCH2 Signaling Pathway and pRB Proteasomal Degradation and the Clinical Relevance of Mucolipin TRPML2 Overexpression in Glioblastoma Patients
Source: Int J Mol Sci. 2022 Jan 8;23(2):688. doi: 10.3390/ijms23020688 (PMC8775570; doi:10.3390/ijms23020688)

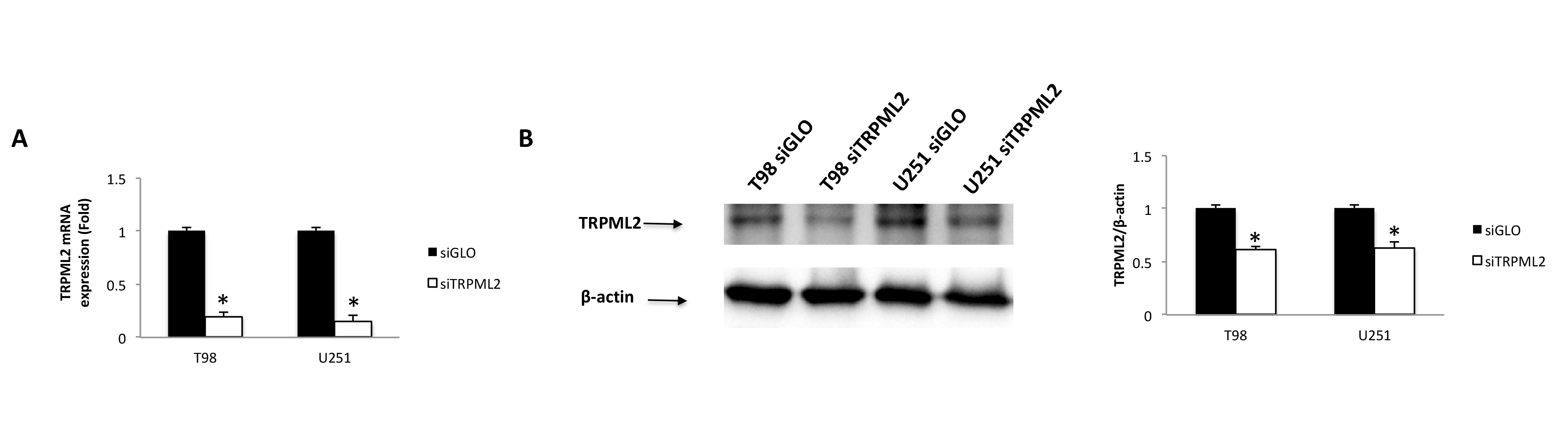

Supplement: Supplementary file 1 [file ijms-23-00688-s001.zip › Supplementary Figures/Supplementary Figure S1 .tif]

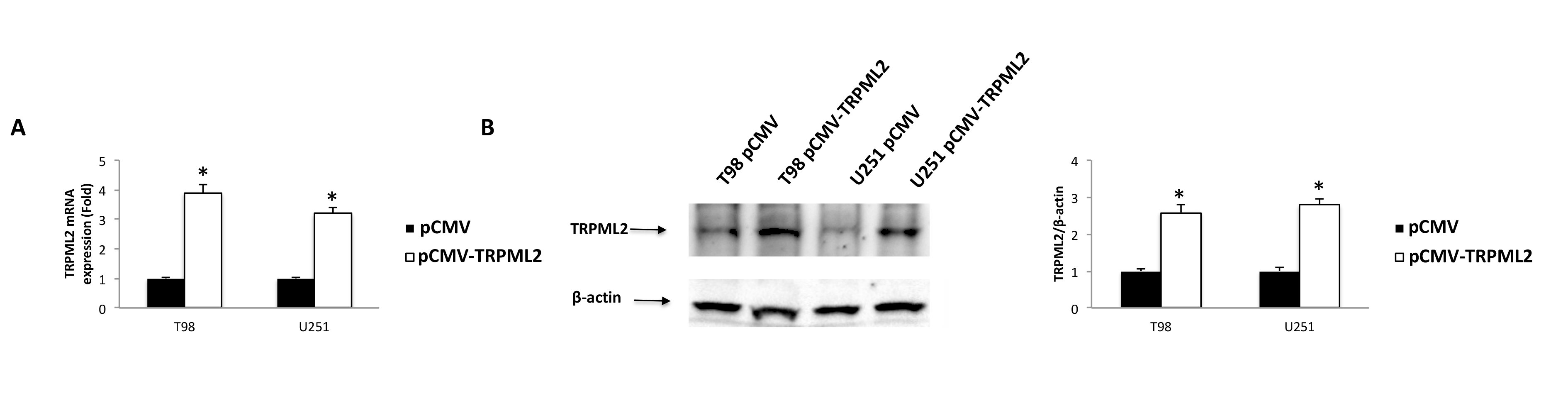

Supplement: Supplementary file 1 [file ijms-23-00688-s001.zip › Supplementary Figures/Supplementary Figure S2.tif]

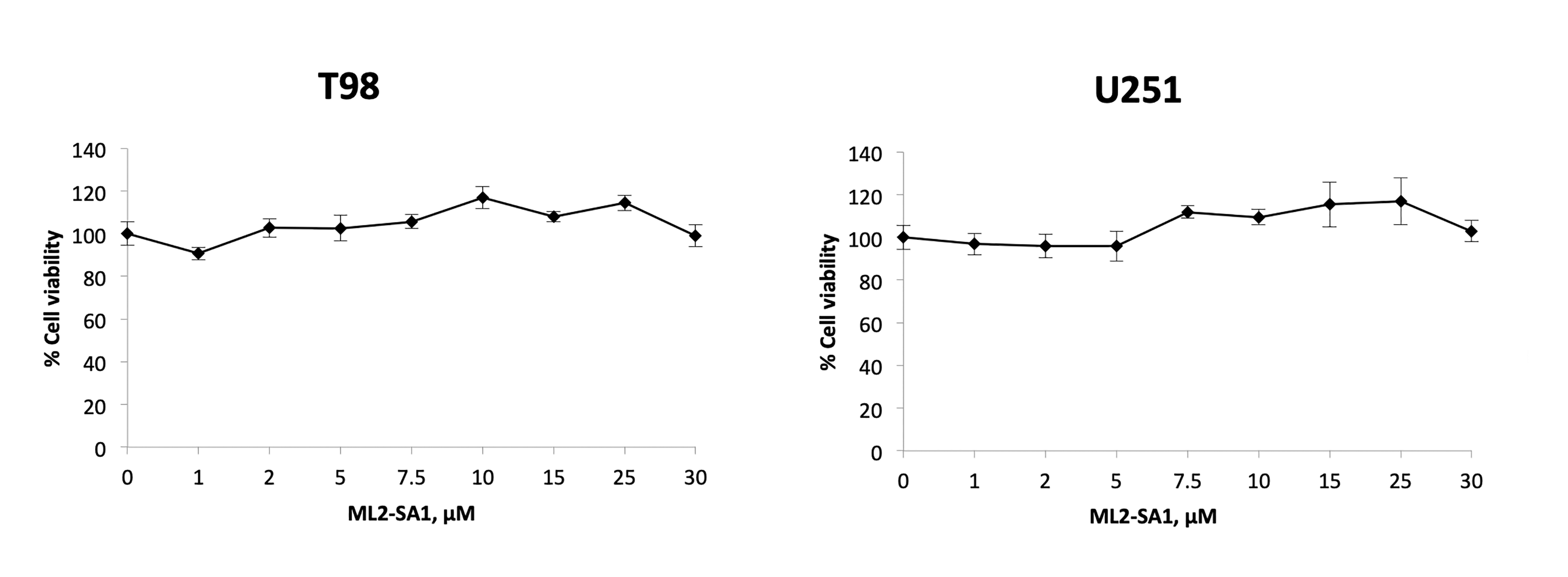

Supplement: Supplementary file 1 [file ijms-23-00688-s001.zip › Supplementary Figures/Supplementary Figure S3.tif]

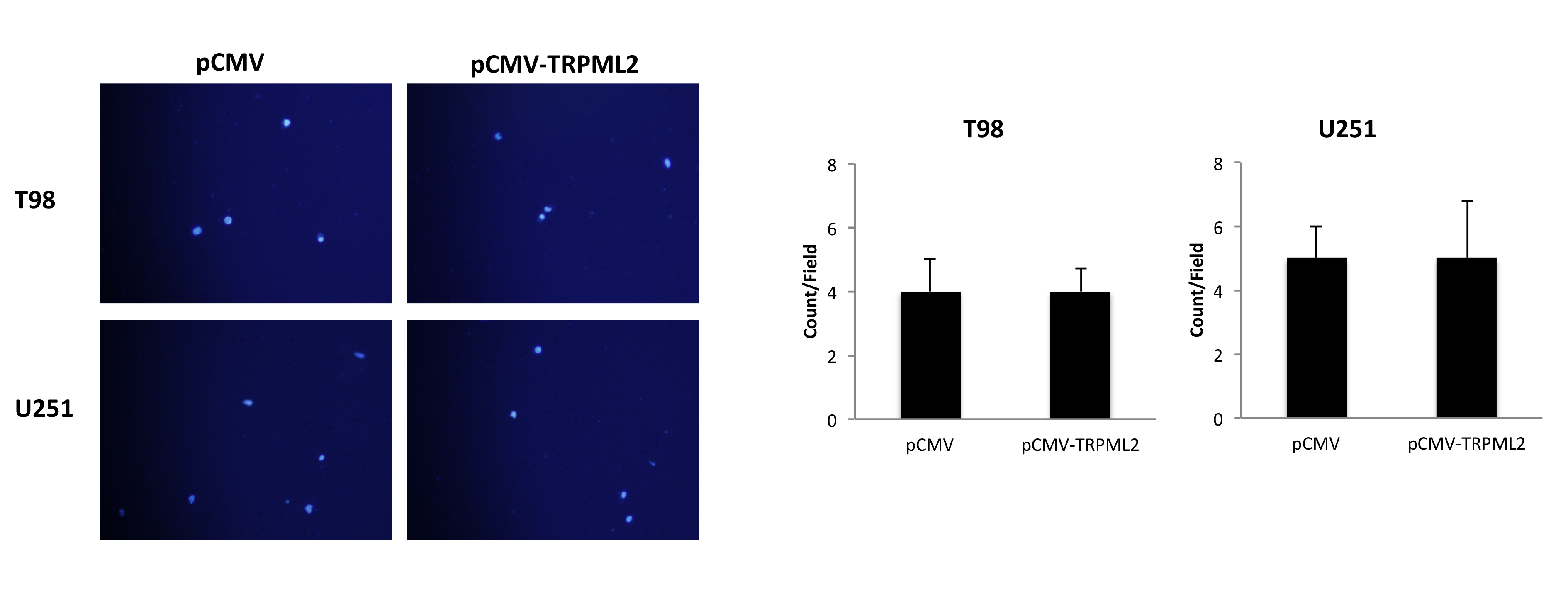

Supplement: Supplementary file 1 [file ijms-23-00688-s001.zip › Supplementary Figures/Supplementary Figure S5.tif]

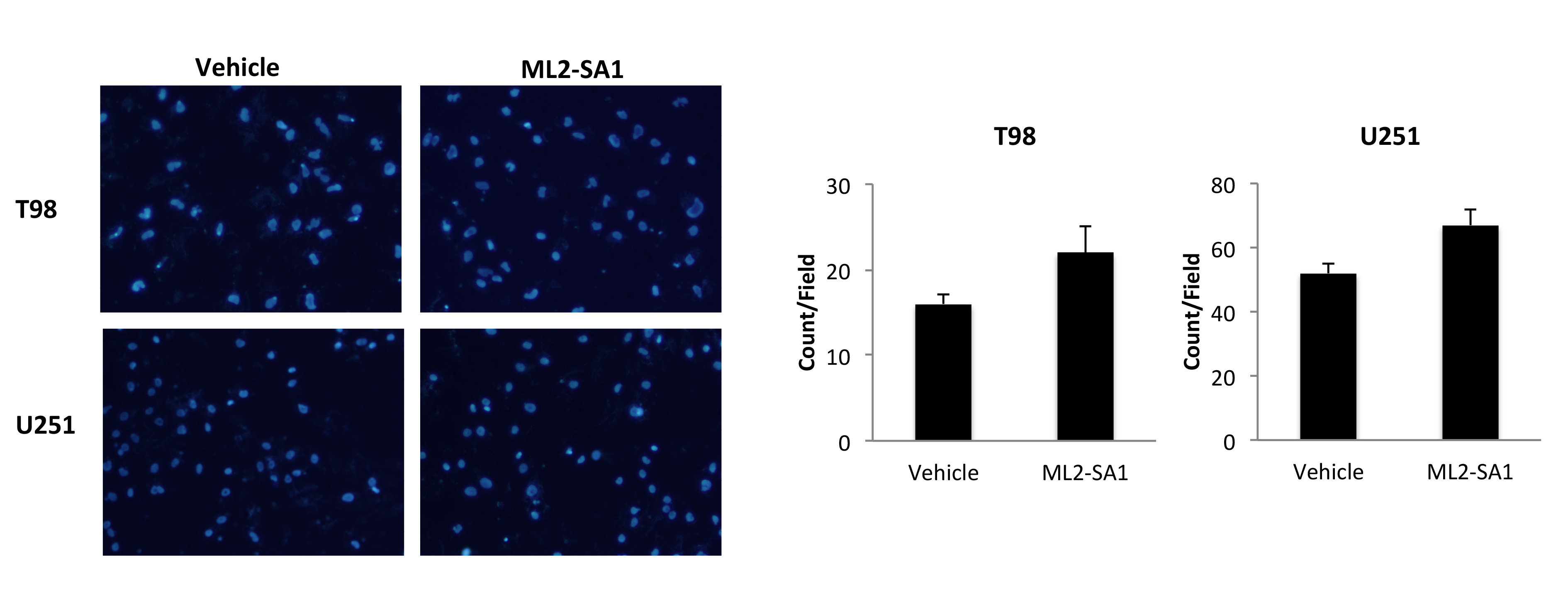

Supplement: Supplementary file 1 [file ijms-23-00688-s001.zip › Supplementary Figures/Supplementary Figure S4 .tif]
